# ACC010, a novel BRD4 inhibitor, synergized with homoharringtonine in acute myeloid leukemia with *FLT3* *‐*ITD

**DOI:** 10.1002/1878-0261.13368

**Published:** 2023-01-21

**Authors:** Yu Qian, Xiang Zhang, Shihui Mao, Wenwen Wei, Xiangjie Lin, Qing Ling, Wenle Ye, Fenglin Li, Jiajia Pan, Yutong Zhou, Yanchun Zhao, Xin Huang, Jiansong Huang, Hongyan Tong, Jie Sun, Jie Jin

**Affiliations:** ^1^ Department of Hematology, The First Affiliated Hospital Zhejiang University School of Medicine Hangzhou China; ^2^ Zhejiang Provincial Key Laboratory of Hematopoietic Malignancy Zhejiang University Hangzhou China; ^3^ Zhejiang Provincial Clinical Research Center for Hematological Disorders Hangzhou China; ^4^ Zhejiang University Cancer Center Hangzhou China; ^5^ The Affiliated People's Hospital of Ningbo University China; ^6^ Jinan Microecological Biomedicine Shandong Laboratory China

**Keywords:** acute myeloid leukemia, *FLT3*‐ITD, homoharringtonine, novel BRD4 Inhibitor ACC010

## Abstract

Bromodomain‐containing protein 4 (BRD4) inhibitors have been clinically developed to treat acute myeloid leukemia (AML), but their application is limited by the possibility of drug resistance, which is reportedly associated with the activation of the WNT/β‐catenin pathway. Meanwhile, homoharringtonine (HHT), a classic antileukemia drug, possibly inhibits the WNT/β‐catenin pathway. In this study, we attempted to combine a novel BRD4 inhibitor (ACC010) and HHT to explore their synergistic lethal effects in treating AML. Here, we found that co‐treatment with ACC010 and HHT synergistically inhibited cell proliferation, induced apoptosis, and arrested the cell cycle in FMS‐like tyrosine kinase 3‐internal tandem duplication (*FLT3*‐ITD)–positive AML cells *in vitro*, and significantly inhibiting AML progression *in vivo*. Mechanistically, ACC010 and HHT cooperatively downregulated MYC and inhibited FLT3 activation. Further, when HHT was added, ACC010‐resistant cells demonstrated a good synergy. We also extended our study to the mouse BaF3 cell line with FLT3‐inhibitor‐resistant *FLT3*‐ITD/tyrosine kinase domain mutations and AML cells without *FLT3*‐ITD. Collectively, our results suggested that the combination treatment of ACC010 and HHT might be a promising strategy for AML patients, especially those carrying *FLT3*‐ITD.

## Graphical abstract

Co‐treatment with a novel BRD4 inhibitor (ACC010) and homoharringtonine (HHT) synergistically inhibited cell proliferation, induced apoptosis, and arrested the cell cycle in AML with *FLT3*‐ITD mutation. Mechanistically, ACC010 and HHT cooperatively downregulated MYC and inhibited FLT3 activation. Further, HHT could enhance the efficacy of the BRD4 inhibitor to help overcome resistance by targeting MYC and WNT/β‐catenin pathway.

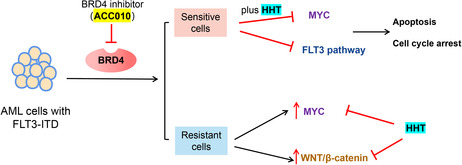

**Abbreviations**
AML: acute myeloid leukemia
BRD4: bromodomain‐containing protein 4
FLT3‐ITD: FMS‐like tyrosine kinase 3‐internal tandem duplication
HHT: homoharringtonine
shRNA: short hairpin RNA
TKD: tyrosine kinase domain
TKI: tyrosine kinase inhibitor
TKI‐R: tyrosine kinase inhibitor resistance

## 1 Introduction

Acute myeloid leukemia (AML) is the most common form of acute leukemia in adults which is characterized by genetic heterogeneity [1]. Under the current therapeutic strategy, which mainly consists of chemotherapy and hematopoietic stem cell transplantation, the outcome remains poor with a higher recurrence rate and higher mortality. Despite the improvement in precision medicine, for new drugs targeting at individual molecular characteristics to benefit some molecular subtypes of AML, primary and secondary drug resistance remains a serious concern for most patients.

FMS‐like tyrosine kinase‐3 (FLT3) is a receptor tyrosine kinase normally expressed in hematopoietic stem and progenitor cells which plays a critical role in early hematopoiesis [2]. *FLT3* internal tandem duplication (*FLT3*‐ITD) is the most common mutational form in AML and occurs in approximately 25% of AML and 30% of cytogenetic normal AML [3, 4]. AML patients with *FLT3*‐ITD have a high incidence of relapse and relatively short survival duration. Current evidence suggests that *FLT3*‐ITD constitutively activates FLT3 kinase activity to promote the proliferation of AML [5]. Although *FLT3*‐ITD alone is not sufficient to generate AML, the role of a therapeutic target has broadened the treatment options for AML patients [6]. Consistently, several FLT3 kinase inhibitors (FLT3‐TKIs), such as gilteritinib and quizartinib, have been clinically developed and exhibited a favorable response in patients with *FLT3‐*ITD/TKD mutations. However, TKI resistance (TKI‐R) may emerge in acquired *FLT3*‐TKD mutations or in the case of high levels of FLT3 ligand [7, 8]. Thus, an alternative therapy is highly needed under such circumstances.

BRD4 belongs to the family of BET (bromodomain and extraterminal) proteins, called chromatin “readers” that bind to the acetylated lysines on histone proteins, which has an important impact on transcriptional regulation of multiple important oncogenes, including *MYC* [9]. BRD4 has been reported to be crucial in AML maintenance through *MYC* activation and aberrant transcriptional elongation [10]. In AML with *FLT3*‐ITD, BRD4 inhibitors, alone or in combination with FLT3 inhibitors, also exhibited excellent proliferation inhibition [11]. To date, several small‐molecule inhibitors of BRD4, such as JQ1, OTX015, and Molibresib, have been developed and some of them have entered phase I/II clinical trials [12, 13]. However, despite their high efficacy in *in vitro* experiments, their clinical outcome is unsatisfactory when administrated as single agents, along with the issue of the development of resistance. It has been reported that the mechanism of BRD4 inhibitor resistance in AML was mainly associated with WNT/β‐catenin–mediated *c‐MYC* reactivation after suppression, so targeting the WNT/β‐catenin–c‐MYC axis possibly could help overcome BRD4 inhibitor resistance [14].

Homoharringtonine (HHT), a classic antileukemia drug in China, was originally isolated from the *Cephalotaxus hainanensis*. Over the past few years, our research team has devoted great efforts to understand its effect and mechanism in AML [15, 16, 17, 18]. In our previous clinical trial, we demonstrated that HHT in combination with cytarabine and aclarubicin achieved a high complete remission rate of 73–83% in treating *de novo* AML [15, 16]. Because this HHT‐based regimen has high efficiency and is inexpensive, it has been the first‐line choice for AML therapy in China. Furthermore, we found that HHT showed high efficiency in AML with *FLT3*‐ITD via downregulating FLT3 expression and inhibiting FLT3‐mediated downstream signaling activation [17]. Moreover, HHT inhibited *c‐MYC* activation by directly binding the NF‐κB‐repressing factor, whereas HHT also reduced the protein expression of CTNNB1, which is known as an intracellular signal transducer in the WNT signaling pathway [19]. Therefore, HHT could be potentially combined with BRD4 inhibitors in *FLT3*‐ITD–positive AML.

Because BRD4 inhibitors and HHT could downregulate MYC through different pathways, and the downregulation of WNT/β‐catenin by HHT presumably could help overcome resistance to BRD4 inhibitors, we attempted to combine a novel BRD4 inhibitor ACC010 and HHT in AML cells. In this study, we demonstrated that the combination of ACC010 and HHT had significant synergistic effects in AML with *FLT3*‐ITD *in vitro* and *in vivo*. The combination inhibited cell proliferation by inducing apoptosis and arrested the cell cycle at the G0/G1 phase. Mechanistically, downregulation of MYC and inhibition of the FLT3 pathway may account for the synergistic effects. Furthermore, synergistic effects were also shown in cells resistant to ACC010 or FLT3 inhibitors. Moreover, the synergistic effects of this combination could also be expanded to some subtypes of *FLT3*‐ITD–negative AML. Therefore, our studies suggested that ACC010 plus HHT might be a promising combination regimen to treat AML, especially *FLT3*‐ITD–positive AML.

## 2 Materials and methods

### 2.1 Agents and antibodies

ACC010 was kindly provided by Jiangsu Aidea Pharmaceutical Co., Ltd. (Yangzhou, China). The synthesis of ACC010 was described in the patent CN106132968B (Fig. S1A–D). ACC010 was dissolved in dimethyl sulfoxide for *in vitro* assays and in 0.5% sodium carboxymethyl cellulose (CMC‐Na) for *in vivo* assays. HHT was purchased from Med Chem Express (Monmouth Junction, NJ, USA) and was dissolved in phosphate‐buffered saline for *in vitro* and *in vivo* assays. JQ1 was purchased from Sigma‐Aldrich (St Louis, MO, USA) and was dissolved in dimethyl sulfoxide. OTX015 was purchased from Selleckchem (Houston, TX, USA) and was dissolved in dimethyl sulfoxide. The antibodies GAPDH, FLT3, p‐FLT3, STAT5, p‐STAT5, PARP, caspase‐3, caspase‐7, CDK2, CDK4, CDK6, P21, P27, RB, p‐RB, AKT, p‐AKT, ERK1/2, p‐ERK1/2, MYC, and APC were purchased from Cell Signaling Technology (Beverly, MA, USA). β‐Actin antibody was purchased from Proteintech (Wuhan, China).

### 2.2 Cell lines and primary cells

*FLT3*‐ITD–positive AML cell lines MV4‐11 (RRID: CVCL_0064) and MOLM13 (RRID: CVCL_2119) were a kind gift from R. Bhatia (City of Hope National Medical Center, Duarte, CA, USA) and were cultured in Iscove's Modified Dulbecco's Medium (IMDM) supplemented with 10% FBS (Gibco, Waltham, MA, USA). THP1 (RRID: CVCL_0006), OCI‐AML2 (RRID: CVCL_1619), OCI‐AML3 (RRID: CVCL_1844), KG1‐α (RRID: CVCL_1824), HL‐60 (RRID: CVCL_0002), U937 (RRID: CVCL_0007), JURKAT (RRID: CVCL_0065), and K562 (RRID: CVCL_0004) cell lines were purchased from the Shanghai Cell Bank of the Chinese Academy of Sciences (Shanghai, China). KASUMI‐1 (RRID: CVCL_0589) cell line was gifted by C. Saijuan (Shanghai Institute of Hematology, Shanghai, China). These cells were cultured in Roswell Park Memorial Institute 1640 (RPMI 1640) medium supplemented with 10% FBS. All human cell lines have been authenticated using STR profiling within the last 3 years and that all experiments were performed with mycoplasma‐free cells. As for human samples, the experiments were undertaken with the understanding and written consent of each subject. Human data have been performed in accordance with the Declaration of Helsinki and have been approved by Clinical Research Ethics Committee of the First Affiliated Hospital, College of Medicine, Zhejiang University (IIT20220135B). Mononuclear cells from AML patient samples (*n* = 16) were obtained from the First Affiliated Hospital of Zhejiang University (Hangzhou, China) between 2021 and 2022, isolated by Ficoll–Hypaque (Sigma‐Aldrich) density gradient centrifugation and cultured in RPMI 1640 medium with 10% FBS. Testing for *FLT3*‐ITD and other genetic mutations was performed at the First Affiliated Hospital of Zhejiang University (Hangzhou, China). Patient characteristics can be found in Table S1.

### 2.3 Cell proliferation assay

Acute myeloid leukemia cell lines were seeded in 96‐well plates with 1–2 × 10^4^ cells/well, and primary AML cells were seeded in 96‐well plates with 1 × 10^5^ cells/well. Cells were treated with variable concentrations of ACC010 and/or HHT for 48 h. Cell viability was assayed by CellTiter‐Lumi™ Luminescent Cell Viability Assay Kit (Beyotime, Shanghai, China) according to the manufacturer's instructions.

### 2.4 Growth curve assay

Cells were seeded in 96‐well plates (5000 cells/well), which were treated with single agents or a combination regimen. MTS solution measuring 10 μL (CellTitre 96; Promega) (5 mg·mL^−1^) was added to each well at every 24 h, varying from 0 to 96 h, and the cells were incubated for an additional 4 h at 37 °C before absorbance was measured at 490 nm.

### 2.5 Cell apoptosis and cell cycle assay

Induction of apoptosis was assessed using a kit obtained from MULTI SCIENCES (Shanghai, China), following the manufacturer's instructions. Cells were collected after treatment with drugs for 48 or 72 h. Apoptotic cells were analyzed by flow cytometry after incubation with Annexin‐V FITC and propidium iodide (PI) for 30 min at room temperature using FACScan™ flow cytometer (Becton Dickinson, Franklin Lakes, NJ, USA). Cell cycle assay was also detected by flow cytometry using PI DNA staining from MULTI SCIENCES. After treatment with drugs for 48 h, the cells were harvested, fixed overnight with 75% ethanol at 4 °C, and then incubated with DNA staining for 30 min at room temperature. Analysis was conducted by FACScan™ flow cytometer (Becton Dickinson).

### 2.6 Western blot analysis

Cells were lysed in RIPA buffer (Thermo Fisher Scientific, Waltham, MA, USA) with protease inhibitor and phosphatase inhibitor cocktail (Thermo Fisher Scientific) on ice for 30 min. Next, the cell lysate was centrifuged at 12 000 ***g*** for 15 min at 4 °C, and a BCA reagent (Thermo Fisher Scientific) was used to determine the protein concentration of the cellular supernatant. Western blotting was performed using 4–12% SDS/PAGE gel and cellular proteins were transferred onto a preactivated PVDF membrane (Millipore, Billerica, MA, USA). Then membranes were blocked with 5% nonfat milk for 1 h and incubated overnight with primary antibodies at 4 °C. Subsequently, the membranes were washed thrice with TBST and incubated with secondary antibodies (Cell Signaling Technology) for 1 h at room temperature. The target proteins were visualized using FDbio‐Femto ECL (Fudebio, Hangzhou, China) and analyzed using image lab™ software (Bio‐Rad, Hercules, CA, USA). The densitometry quantification was performed using the imagej software (NIH, Bethesda, MD, USA).

### 2.7 RNA extraction and real‐time PCR (qRT‐PCR)

Total RNA was extracted from the cells using the TRIzol reagent (TaKaRa, Dalian, China). Reverse transcription was performed using RNA PCR core kit (TaKaRa). Quantitative real‐time PCR was carried out using SYBR Green qPCR mastermix (TaKaRa). Analysis was performed with Bio‐Rad CFX96 (Bio‐Rad). GAPDH was used as internal control. The sequences of the primers were as follows:

*GAPDH* (forward 5′‐GGAGCGAGATCCCTCCAAAAT‐3′; reverse 5′‐GGCTGTTGTCATACTTCTCATGG‐3′).
*MYC* (forward 5′‐GGCTCCTGGCAAAAGGTCA‐3′; reverse 5′‐CTGCGTAGTTGTGCTGATGT‐3′).
*APC* (forward 5′‐AAAATGTCCCTCCGTTCTTATGG‐3′; reverse 5′‐CTGAAGTTGAGCGTAATACCAGT‐3′).
*DVL1* (forward 5′‐GAGGGTGCTCACTCGGATG‐3′; reverse 5′‐GTGCCTGTCTCGTTGTCCA‐3′).
*FZD5* (forward 5′‐CCGTTCGTGTGCAAGTGTC‐3′; reverse 5′‐GAAGCGTTCCATGTCGATGAG‐3′).
*GSK3B* (forward 5′‐AGACGCTCCCTGTGATTTATGT‐3′; reverse 5′‐CCGATGGCAGATTCCAAAGG‐3′).

### 2.8 *In vivo* studies

For AML xenografts, female NCG (NOD/ShiLtJGpt‐Prkdcem26Cd52Il2rgem26Cd22/Gpt) mice, aged 5–6 weeks, were purchased from GemPharmatech Co., Ltd (Nanjing, China) and were raised in the Experimental Animal Center of Zhejiang Chinese Medicine University Laboratory Animal Research Center. Mice were exposed to a 10/14 h light–dark cycle, kept under normal room temperature and fed standard pellet food and tap water. All animal experiments were reviewed and approved by the Institutional Animal Care and Use Committee (Approval No: IACUC‐20210517‐11). And all animal experiments were performed following animal use guidelines and ethical approval. In this study, MOLM13‐luciferase cells (1 × 10^6^) were injected into each mouse via the tail vein. After 6 days, cell engraftment was assessed after intraperitoneal injection of luciferin (Promega). Then mice were randomly assigned to four groups according to the intensity of the luciferin signal by using the IVIS Imaging System (PerkinElmer, Waltham, MA, USA). Each group was treated as follows: 0.5% CMC‐Na (days 6–26, PO), 20 mg·kg^−1^ of ACC010 (days 6–26, PO), 0.5 mg·kg^−1^ of HHT (days 6–12, intraperitoneal), or 20 mg·kg^−1^ of ACC010 and 0.5 mg·kg^−1^ of HHT (administered per the single‐agent group). Mouse body weight was determined once a week. The growth of the leukemia cells was monitored every week by using an IVIS. The survival curve of the mice was analyzed by graphpad prism 9 (San Diego, CA, USA).

### 2.9 Cell transfection

To knock down *BRD4*, short hairpin RNAs (shRNAs) were designed and cloned into a modified pLKO.1‐puro‐shRNA plasmid. The sequence of sh1 was GGAAGTGGAAGAGAATAAA. The sequence of sh2 was GATTACTATAAGATCATTA. To knock down *APC*, MV4‐11 was transfected with mCherry protein containing shRNA or control lentiviruses cloned into pLVX‐shRNA2 vector. The sequence of NC was AGCGTGTAGCTAGCAGAGG. The sequence of sh1 was CCCAGTTTGTTTCTCAAGAAA. The sequence of sh2 was TAATGAACACTACAGATAGAA. For *MYC* overexpression, the coding DNA sequence was cloned into the pCDH1‐MSCV‐MCS‐EF1‐GreenPuro vector and then transfected into MV4‐11. BaF3 cells were stably expressing *FLT3*‐ITD or *FLT3*‐ITD/F691L or *FLT3*‐ITD/D835V utilizing PCDH lentivirus vector containing those mutations.

### 2.10 Statistical analysis

Data were analyzed using graphpad prism 9 and expressed as mean ± SEM. Statistical significance was assessed using two‐tailed Student's *t*‐tests to compare means between two groups (*P* < 0.05 was considered statistically significant). calcusyn software (Biosoft, Cambridge, UK) was used to calculate combination index. Combination index (CI) values less than 1.0 indicate a synergistic interaction of the two agents in the combination. Assessment of compound synergy was also conducted by Bliss scoring utilizing DrugComb online web application tool (https://drugcomb.fimm.fi). The combination effect is additive if the Bliss score equals 0, whereas the combination effect is synergistic if the Bliss score is positive. Survival was analyzed using the Kaplan–Meier method and analyzed using a log rank test.

## 3 Results

### 3.1 Synergistic anti–*FLT3*‐ITD AML effects of ACC010 and HHT *in vitro*

**Fig. 1:**
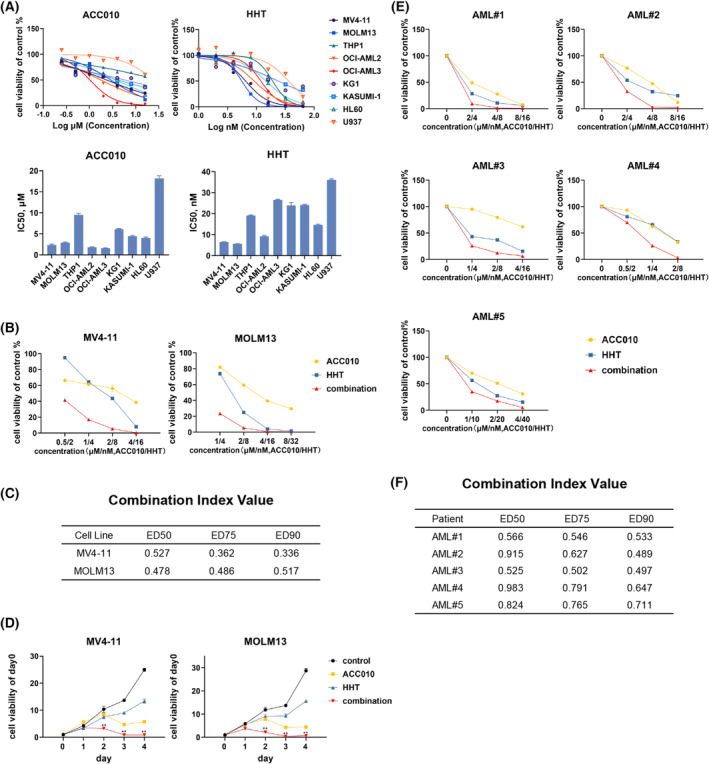
Synergistic anti–*FLT3*‐ITD AML effects of ACC010 and HHT *in vitro*. (A) AML cell lines were treated with variable concentrations of ACC010 or HHT for 48 h and IC50 was shown. Data are presented as mean ± SEM (*n* = 3). (B) MV4‐11 and MOLM13 cells were treated with ACC010 and HHT at a constant ratio for 48 h and cell viability was analyzed. Data are presented as mean ± SEM (*n* = 3). (C) Combination index (CI) values in MV4‐11 and MOLM13 were calculated by calcusyn. (D) Growth curve in MV4‐11 and MOLM13 when added single agent or both in 4 days. Data are presented as mean ± SEM (*n* = 3). Statistical analyses were performed using two‐tailed Student's *t*‐tests. ** *P* < 0.01. (E, F) AML blasts from five patients with *FLT3*‐ITD were cultured with single agent or combination for 48 h to analyze cell viability. The CI values were also presented.

We first evaluated the antileukemia effect of ACC010 and HHT in AML cell lines. We found that both MV4‐11 and MOLM13 cells (carrying *FLT3*‐ITD) were both of the most sensitive to ACC010 and HHT, whereas U937 was relatively resistant to both drugs in AML cell lines (Fig. 1A). Next, we demonstrated that ACC010 and HHT had significant synergistic effects on MV4‐11 and MOLM13, which turned out significant synergistic effect (Fig. 1B,C; Fig. S2A,C). Moreover, both compounds and their combination inhibited the growth of MV4‐11 and MOLM13 cells in a time‐dependent manner (Fig. 1D). These findings showed the synergistic inhibiting effects of ACC010 and HHT in *FLT3*‐ITD AML cell lines. Then we observed the impact of ACC010 and HHT on the viability of primary AML cells in five patients with *FLT3*‐ITD. Primary AML cells demonstrated a good synergy for the combination of ACC010 and HHT (Fig. 1E,F; Fig. S2B,C). We also tested this combination regimen in normal samples from healthy donors or CD34+ hematopoietic stem cells from the umbilical cord blood, and we found limited inhibiting effects in comparison to those in primary AML samples (Fig. S3A). Taken together, the combination of ACC010 with HHT showed a prominent synergistic and highly selective antiproliferative effect in *FLT3*‐ITD–positive AML cells.

### 3.2 ACC010 and HHT synergistically induced the apoptosis and arrested the cell cycle at G0/G1 phase in *FLT3*‐ITD–positive AML cells

**Fig. 2:**
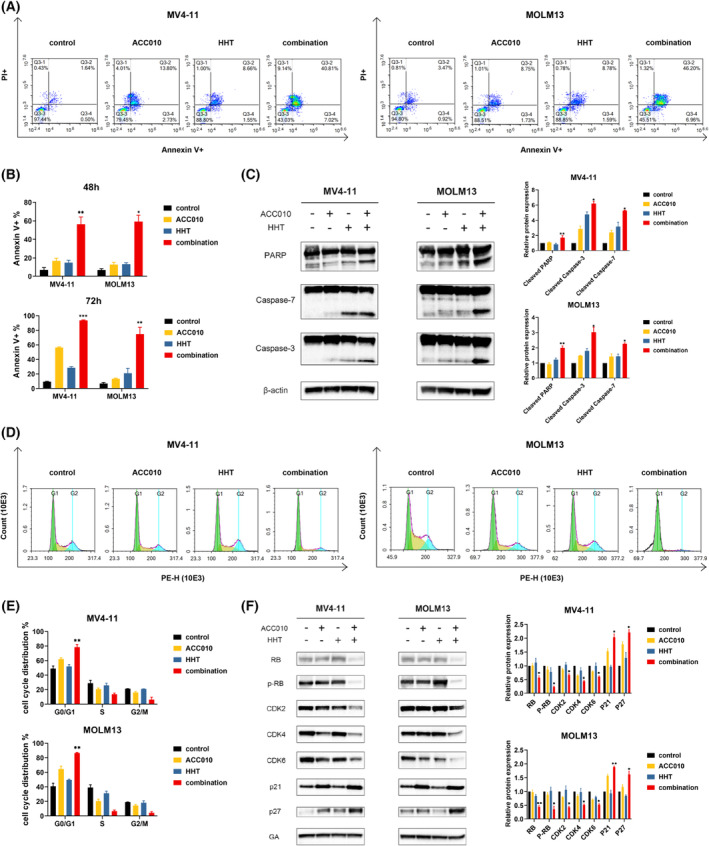
ACC010 and HHT synergistically induced the apoptosis and arrested the cell cycle at G0/G1 phase in *FLT3*‐ITD–positive AML cells. (A) MV4‐11 and MOLM13 were treated with ACC010 2 μm or HHT 4 nm alone or their combination for 48 h, followed by annexin‐V and PI incubation to analyze apoptosis utilizing FCM. (B) Apoptosis in MV4‐11 and MOLM13 induced by two drugs or their combination for 48 and 72 h analyzed by graphpad prism 9. (C) Expression of PARP, Caspase‐7 and Caspase‐3 were analyzed by western blot after treated with ACC010 2 μm or HHT 4 nm alone or their combination for 48 h. (D, E) Cell cycle analysis by flow cytometry in MV4‐11 and MOLM13 cultured with drugs for 48 h. (F) Western blot of CDK2, CDK4, CDK6, RB, p‐RB, p21, and p27 under various administration. Each value indicates the mean ± SEM of three independent experiments (*n* = 3). Statistical analyses were performed using two‐tailed Student's *t*‐tests. *P < 0.05, **P < 0.01, ***P < 0.001.

To further explore their synergy, we investigated the apoptotic phenotype of ACC010 combined with HHT in *FLT3*‐ITD–positive AML cells. We treated both MV4‐11 and MOLM13 cells with 2 μm of ACC010 or 4 nm of HHT or their combination for 48 or 72 h. Annexin‐V and PI staining showed that ACC010 or HHT alone induced cell apoptosis, and the effect was enhanced significantly when ACC010 and HHT were combined (Fig. 2A,B). Besides, the combination of ACC010 and HHT also induced more apoptosis in primary AML cells (Fig. S3B). To confirm the effect of drug‐induced apoptosis, we measured the levels of apoptosis‐related proteins. After treatment with single drugs or a combination regimen for 48 h in MV4‐11 and MOLM13, we extracted the proteins, and the levels of cleaved‐PARP, caspase‐3, caspase‐7 increased more apparently in the combination regimen (Fig. 2C).

Moreover, we also investigated the cell cycle distribution with PI DNA staining under treatment with 2 μm of ACC010, 4 nm of HHT and their combination for 48 h. We observed that 2 μm of ACC010 could induce G0/G1 cell cycle arrest, whereas 4 nm of HHT did not influence the cell cycle. Meanwhile, their combination significantly arrested the cell cycle at the G0/G1 phase compared with the single drug or control group (Fig. 2D,E). Similar results were also found in primary AML cells (Fig. S3C). Further, we found the cell cycle–related proteins to be associated with the phenotype. The protein levels of CDKs (CDK2, CDK4, and CDK6) regulating G1 phase progression decreased to varying degrees in the combination groups. Both RB and pRB protein levels had a greater attenuation in the combined treatments, which implied inhibition of G1/S cell cycle progression. Meanwhile, the levels of P21 and P27 (CDK inhibitors) ameliorated, which also indicated G0/G1 arrest (Fig. 2F). Collectively, ACC010 and HHT inhibited the proliferation of *FLT3*‐ITD–positive AML by the apoptosis induction and G0/G1 cell cycle arrest.

### 3.3 Synergistic antileukemia effect of ACC010 plus HHT on *FLT3*‐ITD–positive AML *in vivo*

**Fig. 3:**
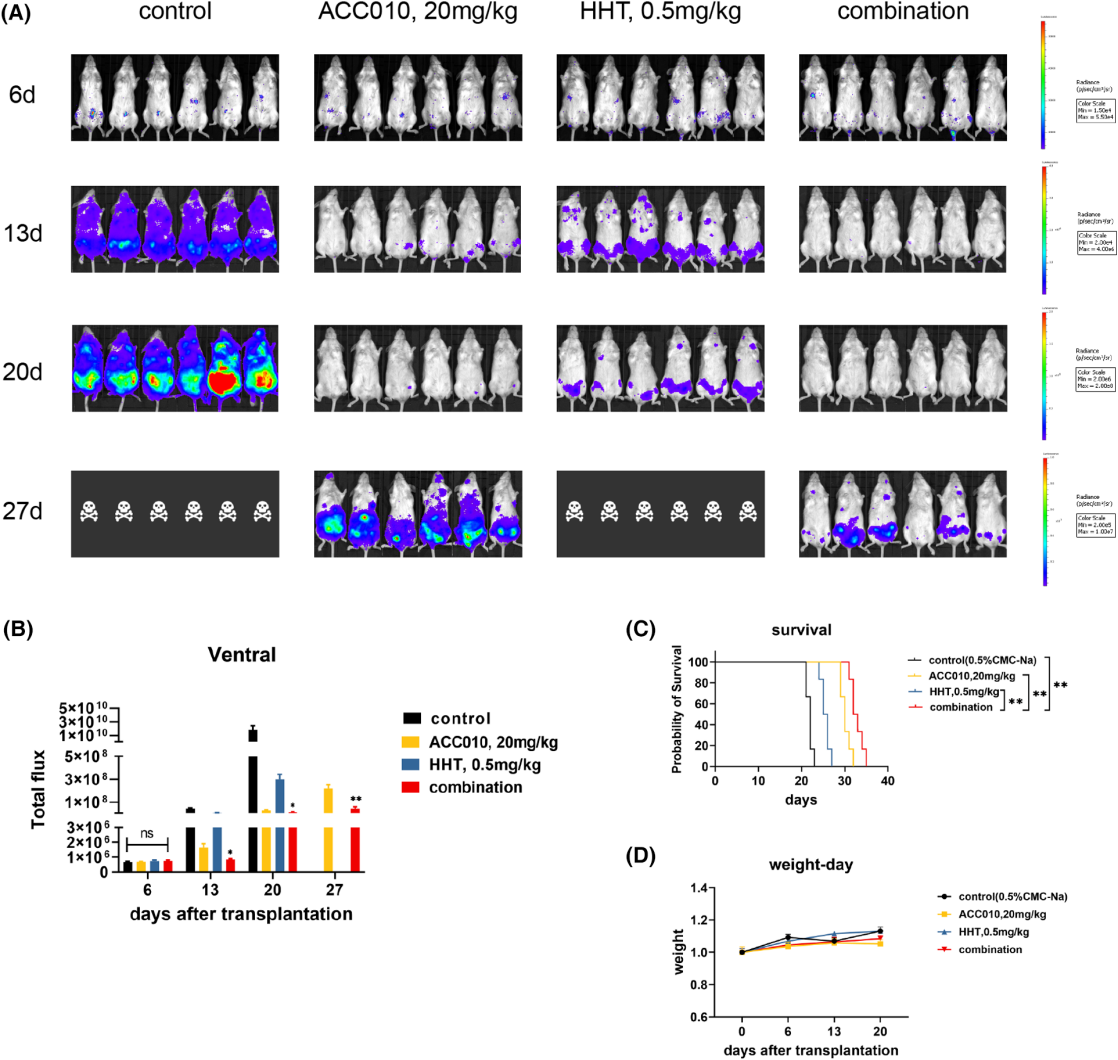
Synergistic antileukemia effect of ACC010 plus HHT on *FLT3*‐ITD–positive AML *in vivo*. (A, B) Leukemia tumor burdens assessed by bioluminescence imaging following treatment and photon intensity was analyzed by graphpad prism 9. Data are presented as mean ± SEM (*n* = 6). (C) Kaplan–Meier survival time for the duration of the treatment (*n* = 6). (D) Body weight change versus time. Statistical analyses were performed using two‐tailed Student's *t*‐tests. *P < 0.05, **P < 0.01.

Owing to their significant synergistic antileukemia effects *in vitro*, we further investigated the effects of ACC010 and HHT *in vivo* in MOLM13‐luc‐NCG xenograft mice. We divided 24 mice into four groups: control group (0.5% CMC‐Na), ACC010 group (20 mg·kg^−1^), HHT group (0.5 mg·kg^−1^), and combination group (20 mg·kg^−1^ of ACC010 and 0.5 mg·kg^−1^ of HHT). We injected each mouse with 1 × 10^6^ MOLM13‐luciferase cells. Six days after injection, when leukemia cells were engrafted in the bone marrow, we started the aforementioned drug administrations. All mice had equal tumor burdens before being administered any treatment. Mice treated with a combination of ACC010 (20 mg·kg^−1^) and HHT (0.5 mg·kg^−1^) had significantly lower leukemia tumor burdens on day 13 and day 20 than mice treated with vehicle or ACC010 or HHT alone (Fig. 3A,B). After 27 days, only mice in the ACC010 group and combination group survived. The latter showed more reduction in tumor burden. All treatment groups had prolonged survival, with the combination group exhibiting the best survival (Fig. 3C). To assess treatment tolerance, we determined the weights of the mice every week. We found the drug doses to be well‐tolerated because there was no obvious effect on body weight (Fig. 3D). Hence, ACC010 and HHT exhibited a synergistic antileukemia effect on *FLT3*‐ITD–positive AML *in vivo*.

### 3.4 ACC010 synergized with HHT to inhibit the proliferation of *FLT3*‐ITD–positive AML via downregulating FLT3‐activated signaling pathway and c‐MYC

**Fig. 4:**
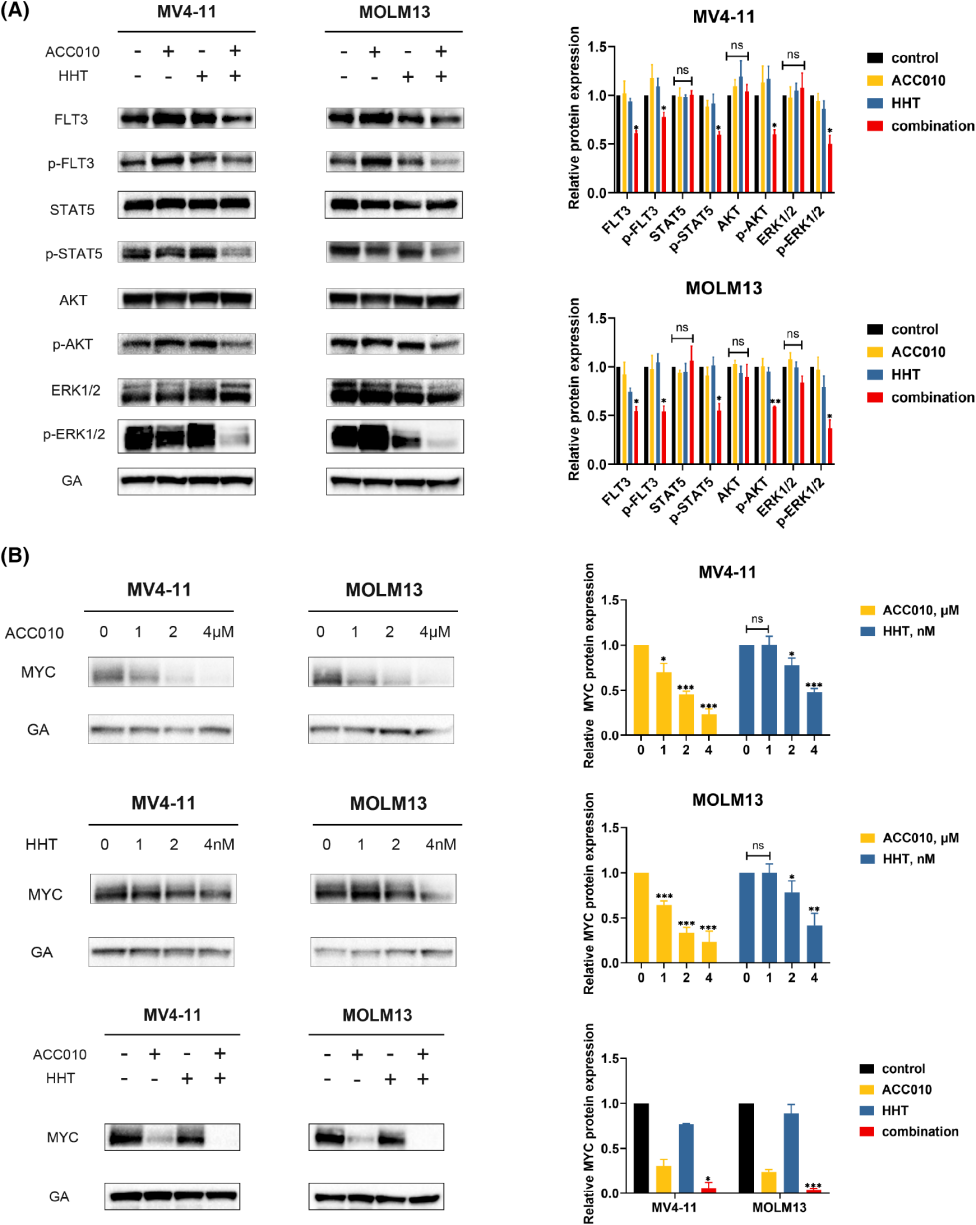
ACC010 synergized with HHT to inhibit the proliferation of *FLT3*‐ITD–positive AML via downregulating FLT3‐activated signaling pathway and c‐MYC. (A) MV4‐11 and MOLM13 were treated with ACC010 2 μm or HHT 4 nm alone or their combination for 48 h. Western blot of FLT3, p‐FLT3, STAT5, p‐STAT5, AKT, p‐AKT, ERK1/2 and p‐ERK1/2 were presented. (B) MYC protein levels after treatment of various concentrations of single agent or combination assessed utilizing western blot. All western blot experiments were repeated three times (*n* = 3). Data are presented as mean ± SEM (*n* = 3). Statistical analyses were performed using two‐tailed Student's *t*‐tests. *P < 0.05, **P < 0.01, ***P < 0.001.

The synergistic effects in MV4‐11 and MOLM13, which express *FLT3*‐ITD, were notable. As reported, ITD mutations lead to the constitutive activation of FLT3 and aberrant activation of multiple downstream pathways such as STAT5, AKT, and ERK1/2 [20]. Thus, we detected the FLT3 and FLT3‐activated signaling pathways after ACC010 and HHT treatments. We observed that the co‐treatment of ACC010 and HHT mediated greater attenuation of FLT3 as well as its activated form p‐FLT3; at the same time, the downstream signaling of FLT3 was also inhibited by this combination, which was revealed by the downregulation of p‐STAT5, p‐AKT and p‐ERK1/2 (Fig. 4A). Thus, ACC010 combined with HHT inhibited the activation of the FLT3‐related pathway.

Meanwhile, previous studies reported that BET inhibitors could deplete the binding of *BRD4* to super‐enhancers, resulting in transcription elongation defects of some oncogenes, including *MYC* [21]. Recently, it has been reported that HHT also targeted MYC via binding NF‐κB‐repressing factor. *MYC* was also critical for the maintenance of *FLT3*‐ITD–positive AML. Thus, we investigated whether BET inhibitors could cooperate to inhibit MYC to repress *FLT3*‐ITD–positive AML. In our study, we observed that both ACC010 and HHT decreased the MYC level in a dose‐dependent manner and that their combination at a lower concentration also decreased the expression of MYC (Fig. 4B). Therefore, we consider that MYC downregulation also accounted for the synergistic effects of ACC010 and HHT in *FLT3*‐ITD–positive AML.

### 3.5 ACC010 and HHT synergistically inhibited the proliferation of *FLT3*‐ITD–positive AML resistant to BRD4 inhibitors

**Fig. 5:**
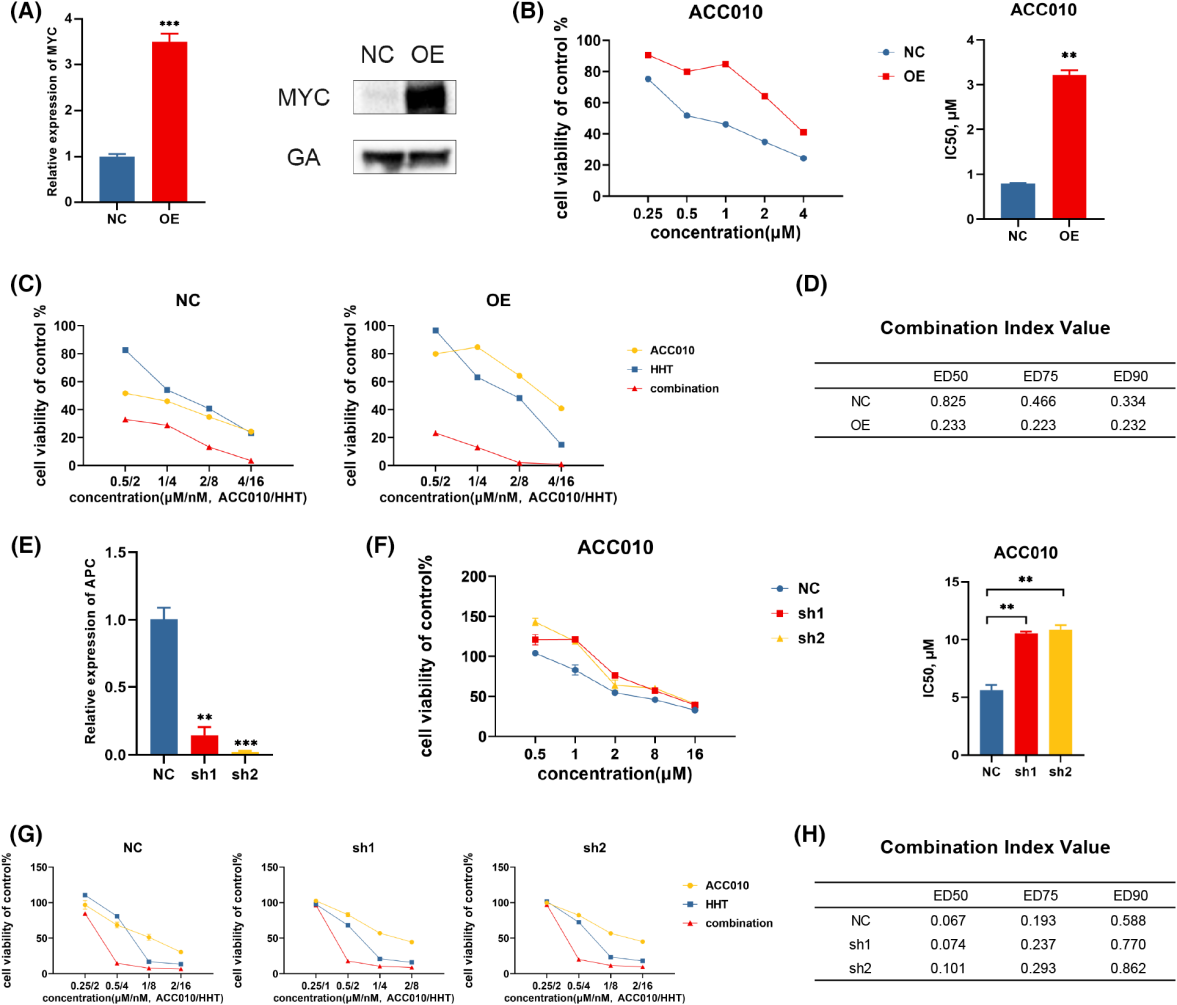
ACC010 and HHT synergistically inhibited the proliferation of *FLT3‐*ITD–positive AML resistant to BRD4 inhibitors. (A) qPCR and western blotting analysis of MYC expression in MV4‐11 with MYC overexpressed or control. Data are presented as mean ± SEM (*n* = 3). (B–D) MV4‐11 with MYC‐OE transfection treated with ACC010 or HHT or both. OE, MYC overexpression; NC, negative control for overexpression. Data are presented as mean ± SEM (*n* = 3). CI values were calculated. (E) qPCR analysis of APC expression in MV4‐11 with different APC shRNAs (sh1, sh2). The NC shRNA was used as a knockdown control. Data are presented as mean ± SEM (*n* = 3). (F–H) APC knockdown in MV4‐11 was analyzed for the IC50 change of ACC010. Data are presented as mean ± SEM (*n* = 3). The synergistic effect of ACC010 and HHT was detected utilizing calcusyn then. Statistical analyses were performed using two‐tailed Student's *t*‐tests. **P < 0.01, ***P < 0.001.

The drug resistance to BRD4 inhibitors remains a critical problem. Previous studies documented that increased expression levels of MYC were related to the resistance in AML. Moreover, whereas BRD4 inhibitors transitorily repressed MYC transcription in types of human leukemias regardless of their sensitivity, resistant cells exhibited a rapid restoration of MYC transcription [22]. In the present study, we selected five cell lines, MV4‐11 and MOLM13 as the cells sensitive to ACC010, and THP1, JURKAT, and K562 as the resistant cells. We observed similar results of finding a rebound of MYC transcription in resistant cells, but not in sensitive cells (Fig. S4A,B). Then we used the MYC‐PCDH lentivirus vector to overexpress *MYC* in MV4‐11, and we discovered that after *MYC* overexpression, MV4‐11 appeared to be more resistant to ACC010 (Fig. 5A,B). The synergistic effect of ACC010 and HHT still existed, and it was more notable than that of the cells infected with overexpression control lentiviral vector (Fig. 5C,D; Fig. S4C).

As has been reported, the activation of the WNT/β‐catenin was the main cause of the restoration of *MYC* transcription, which contributed to the BRD4 inhibitor resistance in AML. The resistant leukemia cell lines showed increased transcription levels of WNT/β‐catenin target genes *DVL1*, *GSK3B*, and *FZD5* [23]. Consistently, we conducted a qRT–PCR analysis of those target genes in AML cell lines and observed that relatively resistant cell lines (THP1, U937, and KG1‐α) were of particularly high expression (Fig. S4D). Literature also supported that knockdown of *APC*, a key molecule that negatively regulates the WNT/β‐catenin, could lead to WNT/β‐catenin activation and resistance to BRD4 inhibitors. Then we transfected MV4‐11 with *APC* shRNA and established *APC* knockdown MV4‐11 cells. Undoubtedly, the *APC* knockdown cells exhibited ACC010 resistance, but an excellent synergistic effect of ACC010 and HHT was still found (Fig. 5E–H; Fig. S4E). Additionally, JURKAT and K562 as ACC010‐resistant leukemia cell lines in our study were also tested by the combination therapy and demonstrated noticeable synergistic effects (Fig. S4F,G). Briefly, the co‐treatment of ACC010 and HHT exhibited excellent synergy in ACC010‐resistant cells.

### 3.6 Co‐treatment of ACC010 and HHT inhibited the proliferation of leukemia cells with *FLT3*‐TKD mutations or without *FLT3*‐ITD

**Fig. 6:**
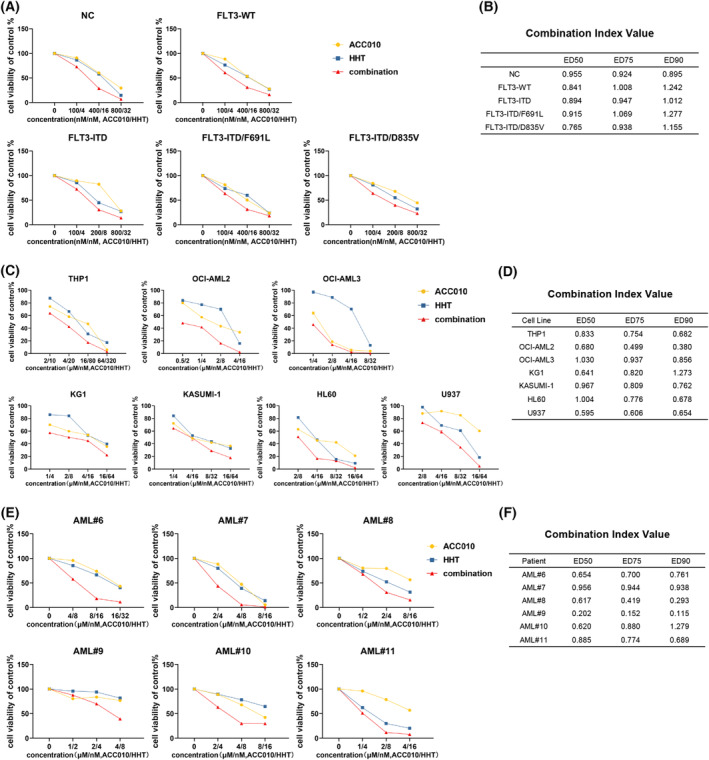
Co‐treatment of ACC010 and HHT inhibited the proliferation of leukemia cells with *FLT3*‐TKD mutations or without *FLT3*‐ITD. (A, B) BaF3 cells transfected with NC (negative control), WT (wild type), FLT3‐ITD, FLT3‐ITD/F691L, and FLT3‐ITD/D835V were treated with ACC010 and HHT at a constant ratio for 48 h respectively. Then cell viability and CI values were analyzed. (C, D) ACC010 and HHT treatment in AML cell lines without *FLT3*‐ITD. Cell viability and CI values were analyzed after 48 h of treatment. (E, F) AML blasts from six patients without *FLT3*‐ITD were treated with single or both drugs for 48 h to analyze cell viability. The CI values were also presented.

ACC010 combined with HHT showed an excellent synergy in *FLT3*‐ITD–positive AML cells, which were also sensitive to FLT3‐TKIs, but whether this combination works in FLT3‐TKI–resistant AML remains undetermined. Previous studies reported that the acquisition of secondary *FLT3*‐TKD mutations including F691L and D835V might account for the resistance to FLT3‐TKIs [8]. As we found that cells with *FLT3*‐ITD mutation had good synergy for ACC010 and HHT, we transfected *FLT3*‐ITD, *FLT3*‐ITD/F691L, and *FLT3*‐ITD/D835V mutations separately into mouse BaF3 cells to further study how *FLT3*‐ITD alone or with *FLT3*‐TKD mutations is affected by the combination therapy. The synergistic effects were shown on both *FLT3*‐ITD– and FLT3‐TKI–resistant cells (Fig. 6A,B; Fig. S4H). Furthermore, we speculated whether this combination could be expanded to AML without *FLT3*‐ITD, so we tested the combination regimen in AML cell lines and AML patients without *FLT3*‐ITD, which resulted in varying degrees of synergistic effects (Fig. 6C–F; Fig. S2A–C). These findings suggest that the co‐treatment of ACC010 and HHT is feasible in treating AML with not only *FLT3*‐ITD but also TKD mutations or without ITD mutation.

### 3.7 HHT synergized with JQ1 or OTX015 to inhibit the proliferation of AML cells

**Fig. 7:**
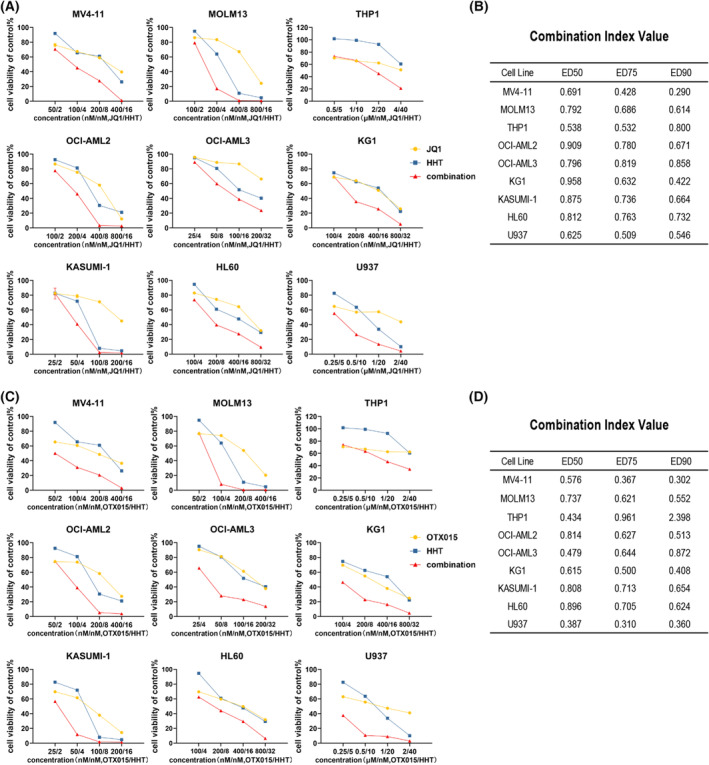
HHT synergized with JQ1 or OTX015 to inhibit the proliferation of AML cells. (A) AML cell lines were treated with JQ1 and HHT at a constant ratio for 48 h and cell viability was analyzed. (B) Combination index (CI) values were calculated by calcusyn. (C, D) OTX015 and HHT treatment in AML cell lines. Cell viability and CI values were analyzed after 48 h of treatment. All experiments were repeated three times (*n* = 3).

Based on the above results, we conjectured that HHT might have synergistic effects with BRD4 inhibitors. Therefore, we conducted the combination regimen of two other BRD4 inhibitors, JQ1 and OTX015, to combine with HHT in AML cell lines. We first evaluated the antileukemia effect of JQ1 and OTX015 in AML cell lines. We found that among these AML cell lines, THP1 and U937 resistant to ACC010, were also relatively resistant to JQ1 and OTX015 (Fig. S5A,B). Then the cell proliferation was tested after treated with single agents or in combination for 48 h. Results confirmed our conjecture that both JQ1 and OTX015 had synergistic inhibiting effects with HHT in AML cells (Fig. 7A–D; Fig. S5C,D). These findings support that the co‐treatment of BRD4 inhibitors and HHT has a great potential for clinical application.

## 4 Discussion

It is noteworthy that targeting BRD4 is a promising strategy in AML therapy. Several structure‐based inhibitors are being developed afterward. Although BRD4 inhibitors showed remarkable antileukemia activity in *in vitro* experiments, the clinical trials were not proceeding as expected owing to limited efficacy and high pharmacokinetics (PK) variability in patients. BRD4 inhibitor OTX015 is the first compound that has undergone a phase I clinical trial in AML (ClinicalTrials.gov, NCT01713582). The results demonstrated that although the clinical response to OTX015 was observed in AML patients, OTX015's therapeutic index (the ratio between its toxicity and therapeutic dose) was narrow. Patients who achieved remissions ultimately relapsed after 2–5 months [12]. Other reports described that BRD4 inhibitor monotherapy may gain resistance due to the restoration of c‐MYC expression induced by WNT/β‐catenin activation. Thus, developing BRD4 inhibitors with a broader therapeutic index and their combinations with other targeted therapy to improve efficacy is of much interest in the future. To date, co‐treatment of BRD4 inhibitors with histone deacetylase inhibitors has exhibited strong synergistic effects in AML with or without FLT3‐ITD [24]. Another combination therapy with DOT1L inhibitors in MLL‐driven AML models has also displayed marked synergistic activity [25]. Our study presented a novel combination regimen of a BRD4 inhibitor and HHT with substantial antileukemia effects *in vitro* and *in vivo*. Furthermore, evidence suggested that MYC provided a possible inherent mechanism for this combination, and HHT might help overcome resistance to BRD4 inhibitors via downregulation of the WNT/β‐catenin pathway. Our results demonstrated that BRD4 inhibitor‐resistant AML cells with *MYC* overexpression or *APC* knockdown were also sensitive to BRD4 inhibitors when combined with HHT.

In this article, we focused on the *FLT3* mutated subtype in AML, which is associated with poor prognosis. Current guidelines recommend earlier incorporation of targeted agents to achieve deeper remissions after detecting FLT3^mut^ at diagnosis. The clinical application of FLT3 inhibitors has improved outcomes in AML patients with *FLT3* mutations. However, as mentioned before, FLT3 inhibitors are faced with a complication that drug resistance compromises their efficacy in clinical therapy. Previous studies reported that resistance mechanisms include the acquisition of secondary *FLT3*‐TKD mutations. Among those mutations, F691 and D835 in *FLT3*‐ITD were found to be substantial barriers to disease control in AML patients treated with FLT3‐TKIs [8]. Those mutations hinder the drug binding sites, making them unfavorable for interaction with FLT3 inhibitors. These findings underscore the need to develop novel agents or to test combination therapies with other agents to improve the outcome of AML with *FLT3*‐ITD in addition to *FLT3*‐TKD mutations.

Currently, some clinical trials involving combination regimens with FLT3‐TKIs in AML have completed, including the combination of sorafenib and 5‐azacytidine, which is proven to be effective in untreated *FLT3*‐ITD–positive AML patients, with an overall response rate of 78% [26]. Another clinical trial to combine sorafenib with histone deacetylase inhibitor vorinostat and proteasome inhibitor bortezomib, and it also observed efficiency in poor‐risk AML [27]. Meanwhile, preclinical studies made several attempts to combine FLT3‐TKIs with other agents to help overcome drug resistance. Among those agents combined with FLT3‐TKIs, BCL2 inhibitor venetoclax exhibited strong synergistic effects on inhibiting the proliferation of *FLT3*‐ITD–positive cells and re‐sensitized FLT3‐TKI–resistant cells; BRD4 inhibitor JQ1 had synergistic lethal effects in AML and could help overcome FLT3‐TKI resistance; HHT could help to inhibit the growth of drug‐resistant clones and increase the antileukemia effects in *FLT3*‐ITD AML [28, 29, 30]. Our study combined BRD4 inhibitor and HHT to treat *FLT3*‐ITD AML. This regimen did not include FLT3‐TKIs but also still presented significant killing effects. In addition, observations suggest that the combination can be utilized in AML cells with *FLT3*‐TKD mutations. Further studies are needed to confirm whether the combination of BRD4 inhibitors and HHT can enhance the efficacy of FLT3‐TKIs to help overcome resistance. Another FLT3‐TKI resistance mechanism was considered to be related to high levels of FLT3 ligand, which could lead to the activation of the FLT3‐MAPK pathway and thus confer resistance to FLT3 inhibitors [7]. This mechanism was not discussed in our article.

## 5 Conclusions

Our study showed a significant synergistic effect of the combination of BRD4 inhibitors and HHT in AML with *FLT3*‐ITD mutation. With *MYC* overexpression or *APC* knockdown to generate cells resistant to BRD4 inhibitor, we found that HHT could enhance the efficacy of the BRD4 inhibitor to help overcome resistance. Furthermore, we extended this regimen to cells with *FLT3*‐TKD mutation and cells without *FLT3*‐ITD. The experimental results revealed the potential of ACC010 combination with HHT in the therapy of AML patients harboring *FLT3*‐TKD who did not respond well to classic FLT3 inhibitors. Meanwhile, the combination regimen could treat AML patients without *FLT3*‐ITD mutation. Moreover, our results support that other BRD4 inhibitors to combine with HHT for AML therapy.

## Conflict of interest

The authors declare no conflict of interest.

## Author contributions

YQ, XZ, and SHM performed the research. JJ designed the research study and supervised the experiments. XH, JSH, HYT, and JS provided specific experimental advice, technical support, and reagents. WWW, XJL, QL, WLY, FLL, JJP, YTZ, and YCZ collected clinical samples and analyzed the data. YQ and XZ wrote the paper. JJ reviewed the paper. All authors read and approved the final manuscript.

### Peer Review

The peer review history for this article is available at https://publons.com/publon/10.1002/1878‐0261.13368.

## Supporting information


**Fig. S1.** Chemical structure of ACC010 and the target protein in AML cell line. A. Chemical structure of ACC010 described in the patent CN106132968B. B. Expression of BRD4, MYC, CDK6 and BCL2 were analyzed by Western blot after treated with ACC010 or JQ1 in MV4‐11. C‐D. The IC50 of ACC010 was analyzed in MV4‐11 BRD4 knockdown cell. ** for p < 0.01, *** for p < 0.001.

**Fig. S2.** Algebraic estimate and Bliss score analysis in AML cell lines and patients. A. The combination index (CI) of ACC010 and HHT in AML cell lines was calculated using CalcuSyn software after 48 hours of drugs’ treatment. B. The combination index (CI) of ACC010 and HHT in AML patients was calculated using CalcuSyn software after 48 hours of drugs’ treatment. C. The Bliss score analysis of combinations in AML cell lines and patients was conducted utilizing DrugComb online web application tool (https://drugcomb.fimm.fi).

**Fig. S3.** Lethal effects of ACC010 and HHT against normal samples and effects on apoptosis and cell cycle in primary AML cells. A. normal samples which from healthy donors or CD34+ hematopoietic stem cells were treated with variable concentrations of ACC010 or HHT for 48 hours and cell viability was analyzed. B. Apoptosis in primary AML cells induced by two drugs or their combination utilizing FCM after incubation with Annexin‐V and PI. C. Cell cycle analysis by flow cytometry in primary AML cells cultured with drugs for 48 h.

**Fig. S4.** Synergistic effects of ACC010 and HHT on ACC010‐resistant leukemia cells and FLT3‐ITD/TKD BaF3 cell. A. AML cell lines were treated with ACC010 for 48 hours and IC50 were showed. B. MYC mRNA levels in indicated leukemia cell lines after 2 h, 6 h, 12 h, 24 h and 48 h of ACC010 treatment (2 μM), relative to DMSO‐treated cells. C. Bliss score analysis of combinations in MY4‐11 MYC‐OE cells. D. mRNA levels of WNT/β‐catenin pathway genes was detected in AML cell lines. The bars represented average of ACC010 resistant AML cell lines (THP1, U937 and KG1‐α) or sensitive cells (MV4‐11, MOLM13, OCI‐AML2, OCI‐AML3, KASUMI‐1, HL60). * for p < 0.05. E. Bliss score analysis of combinations in MY4‐11 APC‐knockdown cells. F‐G. JURKAT and K562 cells were treated with variable concentrations of ACC010 or HHT for 48 hours. Cell viability, CI value and Bliss score were analyzed then. H. Bliss score analysis of combinations in BaF3 cells transfected with NC, WT, FLT3‐ITD, FLT3‐ITD/F691L and FLT3‐ITD/D835V.

**Fig. S5.** JQ1 and OTX015 combined with HHT in AML cell lines. A. AML cell lines were treated with JQ1 for 48 hours and IC50 were showed. B. AML cell lines were treated with OTX015 for 48 hours and IC50 were showed. C. Bliss score analysis of co‐treatment with JQ1 and HHT in AML cell lines utilizing DrugComb online web application tool (https://drugcomb.fimm.fi). D. Bliss score analysis of co‐treatment with OTX015 and HHT in AML cell lines utilizing DrugComb online web application tool (https://drugcomb.fimm.fi).

**Table S1.** Characteristics of primary AML patients.

## Data Availability

All data are available in the manuscript and [Link], [Link], [Link], [Link], [Link].
