# Supplementary figures and images for: ACC010, a novel BRD4 inhibitor, synergized with homoharringtonine in acute myeloid leukemia with FLT3 ‐ITD
Source: Mol Oncol. 2023 Jan 21;17(7):1402–18. doi: 10.1002/1878-0261.13368 (PMC10323884; doi:10.1002/1878-0261.13368)

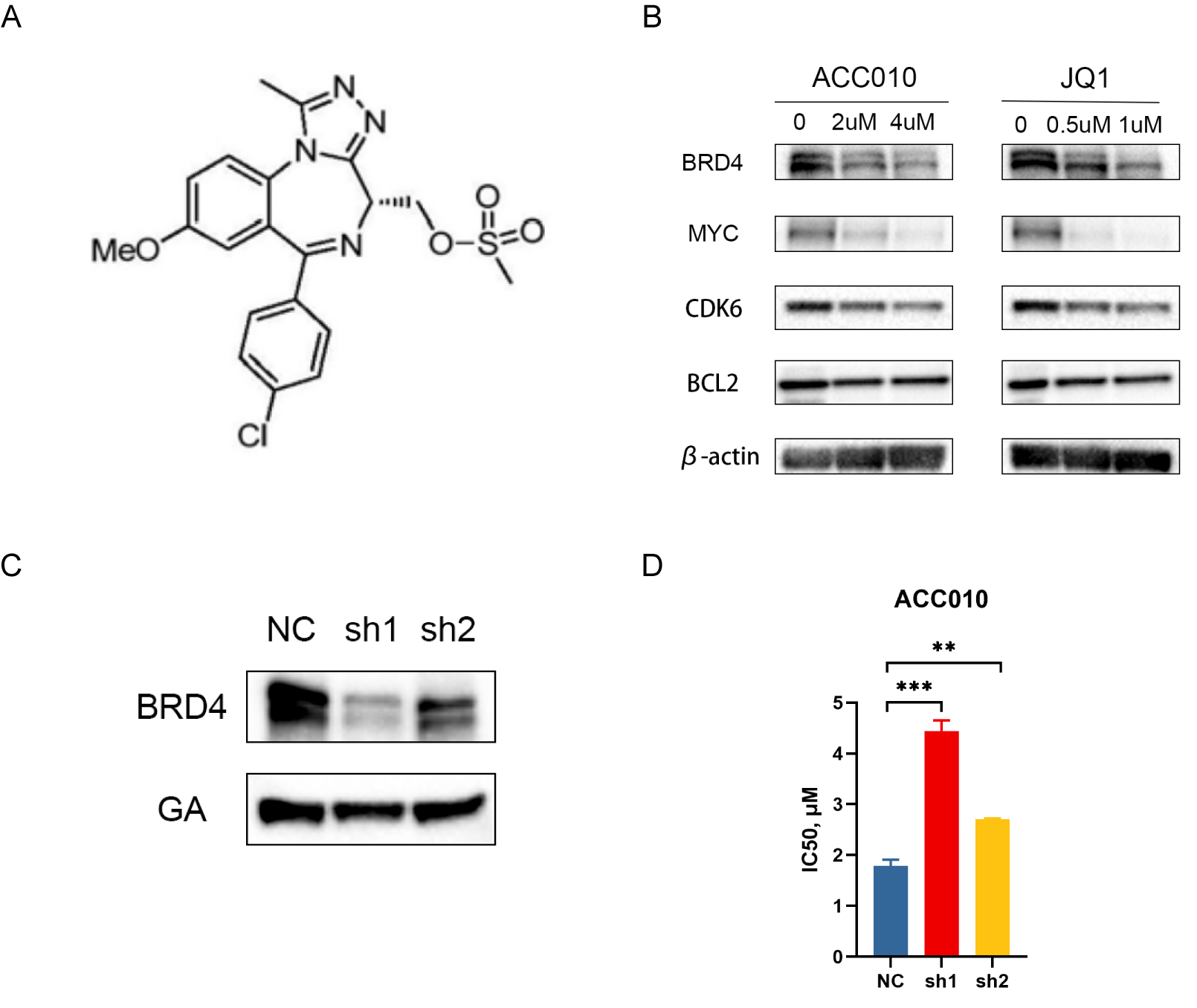

Supplement: Supplementary file 1 — Fig. S1. Chemical structure of ACC010 and the target protein in AML cell line. A. Chemical structure of ACC010 described in the patent CN106132968B. B. Expression of BRD4, MYC, CDK6 and BCL2 were analyzed by Western blot after treated with ACC010 or JQ1 in MV4‐11. C‐D. The IC50 of ACC010 was analyzed in MV4‐11 BRD4 knockdown cell. ** for p < 0.01, *** for p < 0.001. [file MOL2-17-1402-s006.tif]

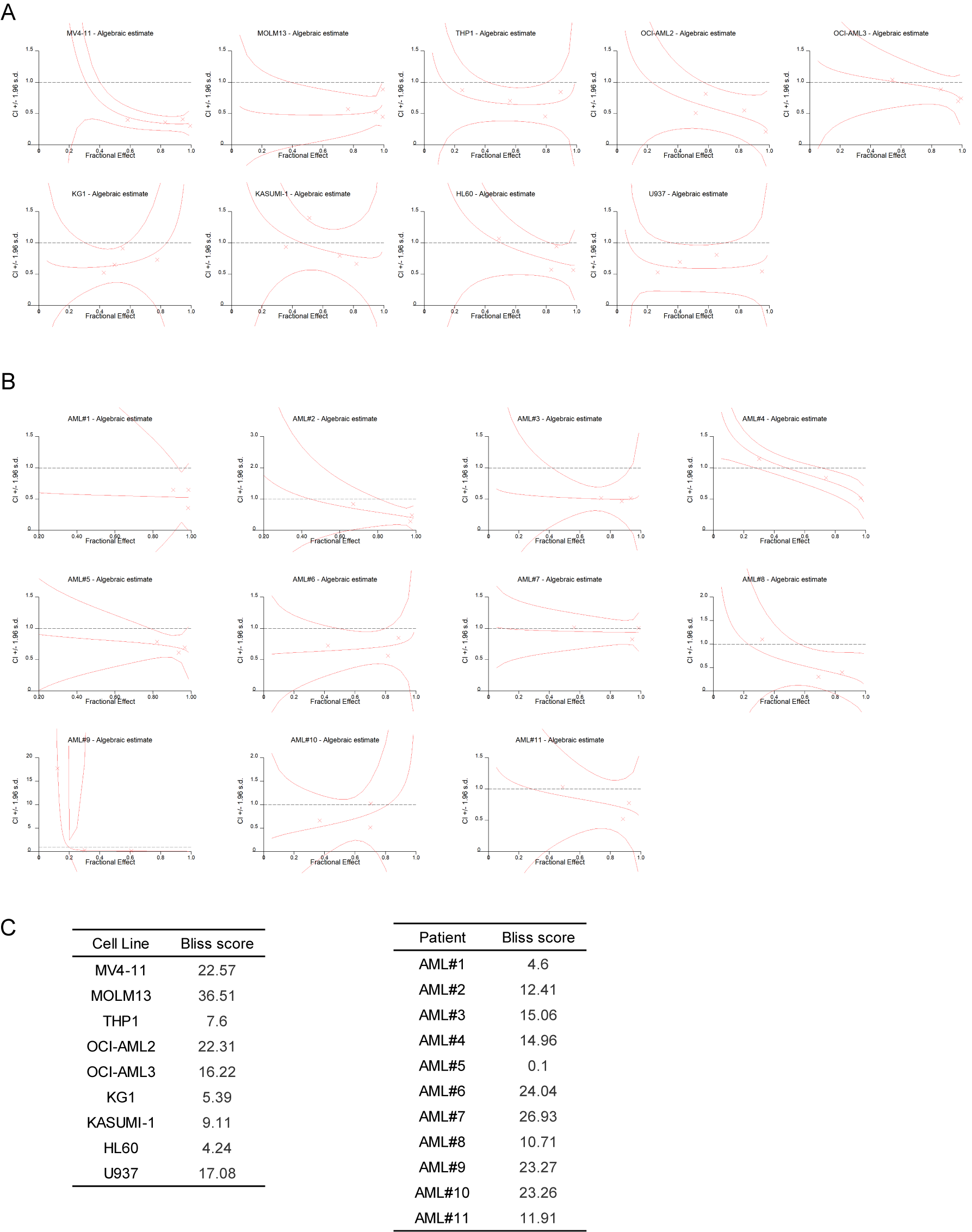

Supplement: Supplementary file 2 — Fig. S2. Algebraic estimate and Bliss score analysis in AML cell lines and patients. A. The combination index (CI) of ACC010 and HHT in AML cell lines was calculated using CalcuSyn software after 48 hours of drugs’ treatment. B. The combination index (CI) of ACC010 and HHT in AML patients was calculated using CalcuSyn software after 48 hours of drugs’ treatment. C. The Bliss score analysis of combinations in AML cell lines and patients was conducted utilizing DrugComb online web application tool (https://drugcomb.fimm.fi). [file MOL2-17-1402-s003.tif]

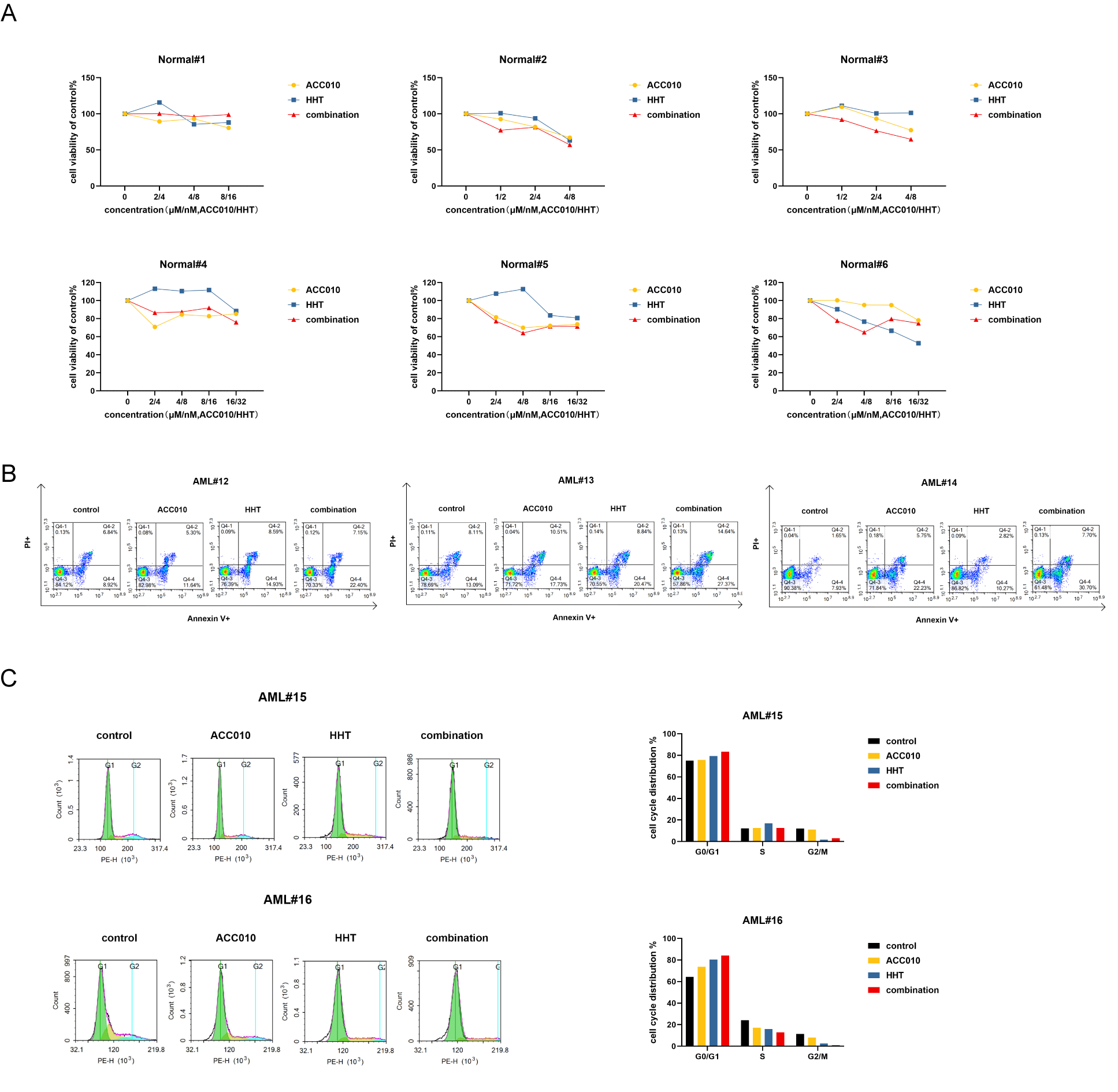

Supplement: Supplementary file 3 — Fig. S3. Lethal effects of ACC010 and HHT against normal samples and effects on apoptosis and cell cycle in primary AML cells. A. normal samples which from healthy donors or CD34+ hematopoietic stem cells were treated with variable concentrations of ACC010 or HHT for 48 hours and cell viability was analyzed. B. Apoptosis in primary AML cells induced by two drugs or their combination utilizing FCM after incubation with Annexin‐V and PI. C. Cell cycle analysis by flow cytometry in primary AML cells cultured with drugs for 48 h. [file MOL2-17-1402-s005.tif]

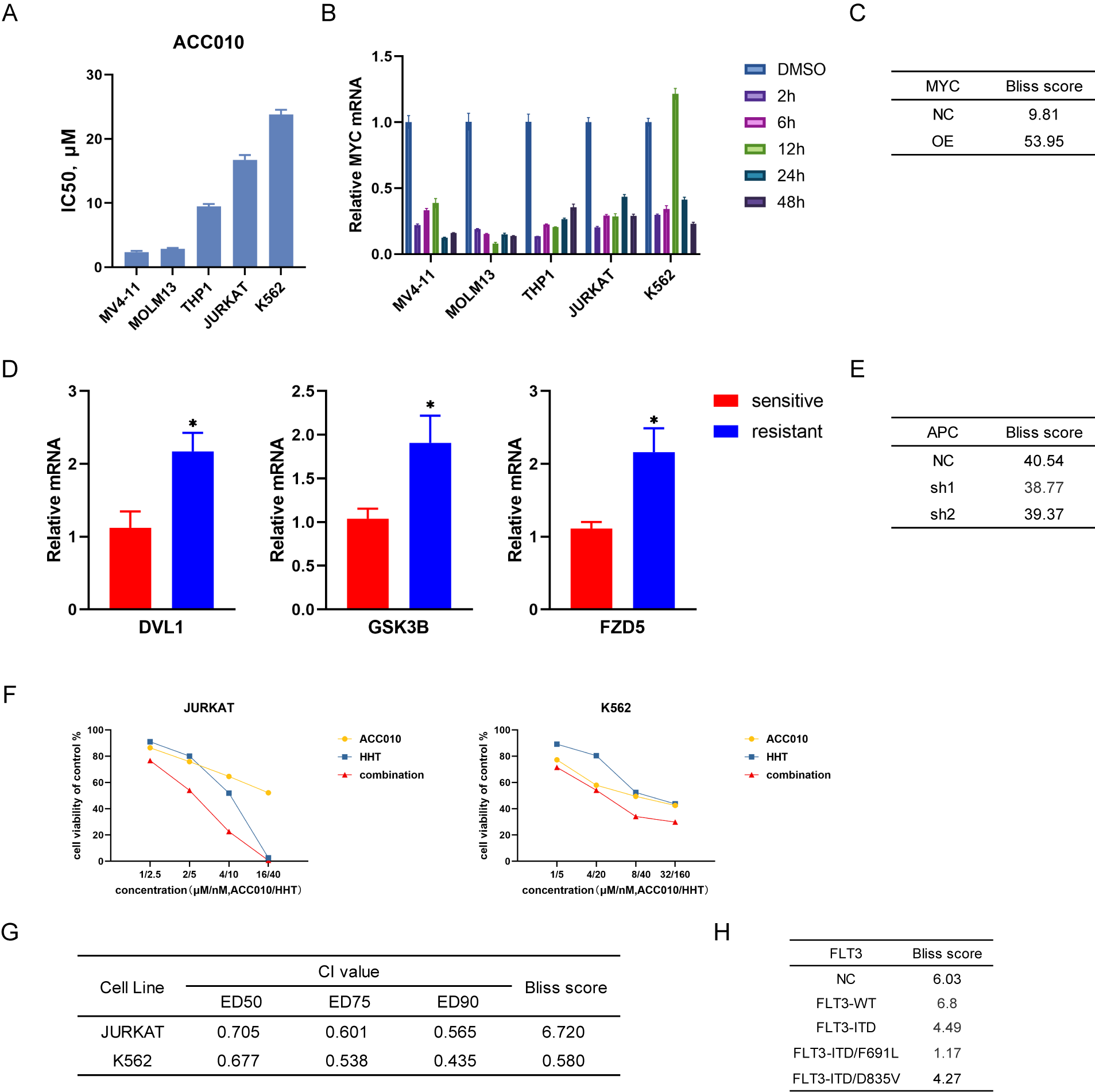

Supplement: Supplementary file 4 — Fig. S4. Synergistic effects of ACC010 and HHT on ACC010‐resistant leukemia cells and FLT3‐ITD/TKD BaF3 cell. A. AML cell lines were treated with ACC010 for 48 hours and IC50 were showed. B. MYC mRNA levels in indicated leukemia cell lines after 2 h, 6 h, 12 h, 24 h and 48 h of ACC010 treatment (2 μM), relative to DMSO‐treated cells. C. Bliss score analysis of combinations in MY4‐11 MYC‐OE cells. D. mRNA levels of WNT/β‐catenin pathway genes was detected in AML cell lines. The bars represented average of ACC010 resistant AML cell lines (THP1, U937 and KG1‐α) or sensitive cells (MV4‐11, MOLM13, OCI‐AML2, OCI‐AML3, KASUMI‐1, HL60). * for p < 0.05. E. Bliss score analysis of combinations in MY4‐11 APC‐knockdown cells. F‐G. JURKAT and K562 cells were treated with variable concentrations of ACC010 or HHT for 48 hours. Cell viability, CI value and Bliss score were analyzed then. H. Bliss score analysis of combinations in BaF3 cells transfected with NC, WT, FLT3‐ITD, FLT3‐ITD/F691L and FLT3‐ITD/D835V. [file MOL2-17-1402-s004.tif]

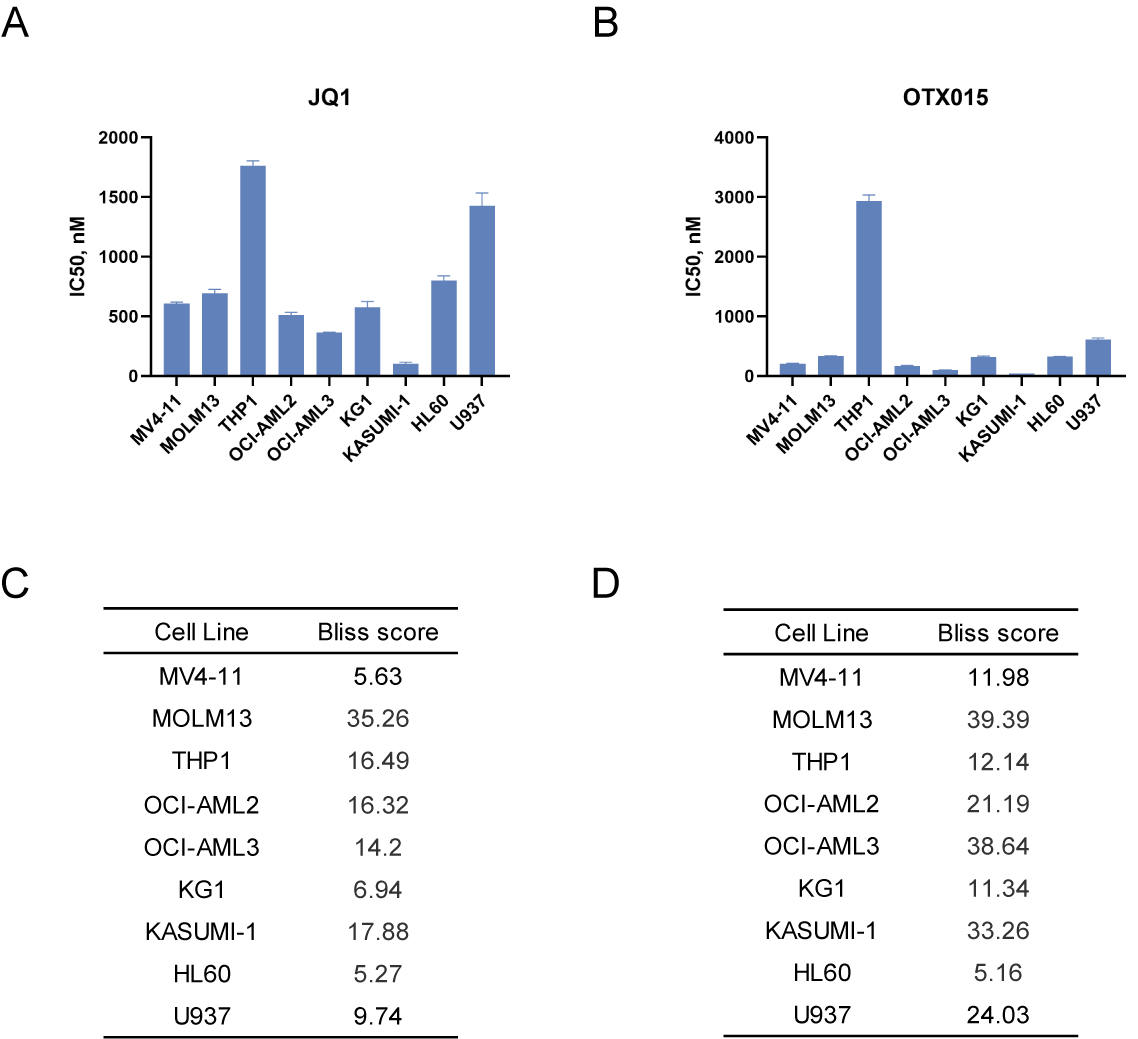

Supplement: Supplementary file 5 — Fig. S5. JQ1 and OTX015 combined with HHT in AML cell lines. A. AML cell lines were treated with JQ1 for 48 hours and IC50 were showed. B. AML cell lines were treated with OTX015 for 48 hours and IC50 were showed. C. Bliss score analysis of co‐treatment with JQ1 and HHT in AML cell lines utilizing DrugComb online web application tool (https://drugcomb.fimm.fi). D. Bliss score analysis of co‐treatment with OTX015 and HHT in AML cell lines utilizing DrugComb online web application tool (https://drugcomb.fimm.fi). [file MOL2-17-1402-s001.tif]
