# Supplementary material for: ACC010, a novel BRD4 inhibitor, synergized with homoharringtonine in acute myeloid leukemia with FLT3 ‐ITD
Source: Mol Oncol. 2023 Jan 21;17(7):1402–18. doi: 10.1002/1878-0261.13368 (PMC10323884; doi:10.1002/1878-0261.13368)
Supplement: Supplementary file 6 — Table S1. Characteristics of primary AML patients. [file MOL2-17-1402-s002.docx]

**Supplementary Tables**

**Table S1. Characteristics of primary AML patients.**

|  | Diagnose | Gender/Age | FAB type | Peripheral blood | BM blast% | Karyotype | Fusion | Mutation |
| --- | --- | --- | --- | --- | --- | --- | --- | --- |
| AML#1 | *de novo* | F/60 | M5b | WBC 25.5x10^9^/L, HB 101g/L, PLT 41x10^9^/L | 84 | NA | NA | FLT3-ITD,  WT1 |
| AML#2 | *de novo* | F/77 | M2 | WBC 98x10^9^/L, HB 51g/L, PLT 67x10^9^/L | 77 | NA | NA | FLT3-ITD,  CEBPA, NPM1 |
| AML#3 | *de novo* | F/55 | M5 | WBC 178.7x10^9^/L, HB 93g/L, PLT 213x10^9^/L | 67 | NA | NA | FLT3-ITD,  DNMT3A, MYC |
| AML#4 | refractory | M/80 | M2/M4 | WBC 86x10^9^/L, HB 78g/L, PLT 51x10^9^/L | 85 | NA | NA | FLT3-ITD |
| AML#5 | refractory | F/21 | M4b | WBC 128x10^9^/L, HB 70g/L, PLT 269x10^9^/L | 60 | NA | MLL-ELL | FLT3-ITD |
| AML#6 | *de novo* | M/71 | M5b | WBC 1.3x10^9^/L, HB 88g/L, PLT 107x10^9^/L | 58 | NA | NA | DNMT3A,  IDH2, PHF6 |
| AML#7 | *de novo* | M/63 | M5b | WBC 199.4x10^9^/L, HB 55g/L, PLT 26x10^9^/L | 77 | NA | CBFB-MYH11 | NA |
| AML#8 | *de novo* | M/43 | M5b | WBC 20.2x10^9^/L, HB 67g/L, PLT 17x10^9^/L | 63.5 | 46, XY[20] | AML1-ETO | TET2, EZH2  PHF6, ASXL1 |
| AML#9 | *de novo* | M/41 | M5b | WBC 54.5x10^9^/L, HB 72g/L, PLT 69x10^9^/L | 47 | 46, XY[20] | NA | NA |
| AML#10 | *de novo* | M/39 | M5b | WBC 104x10^9^/L, HB 164g/L, PLT 47x10^9^/L | 86 | NA | MLL-AF9 | NA |
| AML#11 | refractory | M/50 | M5b | WBC 193.7x10^9^/L, HB 46g/L, PLT 9x10^9^/L | 75 | 46,XY,der(7)(p22),del(9)  (q13q31)[5]/46,XY[5] | NA | NPM1,  NRAS, DNMT3A |
| AML#12 | *de novo* | M/58 | AML | WBC 29.9x10^9^/L, HB 63g/L, PLT 32x10^9^/L | 71.5 | NA | NA | GATA2,  NPM1, TET2 |
| AML#13 | *de novo* | M/30 | M2a | WBC 84.7x10^9^/L, HB 98g/L, PLT 22x10^9^/L | 47 | 45,XY-17[3]/46,XY[7] | NA | FLT3-ITD, CEBPA, GATA2, RAD21 |
| AML#14 | *de novo* | M/46 | M2 | WBC 34.7x10^9^/L, HB 116g/L, PLT 17x10^9^/L | 66 | 46,XY[20] | NA | CEBPA, IKZF1, NRAS, CUX1, GATA2 |
| AML#15 | *de novo* | M/64 | M2a | WBC 61.2x10^9^/L, HB 75g/L, PLT 55x10^9^/L | 85 | NA | NA | DNMT3A, FLT3-ITD, WT1 |
| AML#16 | *de novo* | M/43 | M5b | WBC 40.3x10^9^/L, HB 91g/L, PLT 53x10^9^/L | 76 | NA | NA | FLT3-ITD, MYC |
